# A Public Health Review into Two Decades of Domestic Trampoline Injuries in Children within Queensland, Australia

**DOI:** 10.3390/ijerph20031742

**Published:** 2023-01-18

**Authors:** David Eager, Shilei Zhou, Ruth Barker, Jesani Catchpoole, Lisa N. Sharwood

**Affiliations:** 1 Faculty of Engineering and Information Technology, University of Technology Sydney, Sydney 2007, Australia; 2 Queensland Injury Surveillance Unit, Jamieson Trauma Institute, Royal Brisbane and Women’s Hospital, Herston 4029, Australia; 3 Faculty of Medicine and Health, University of Sydney, Sydney 2006, Australia; 4 Faculty of Medicine and Health, University of New South Wales, Sydney 2032, Australia; 5 Translational Health Collective, Kolling Institute, Clinical School Northern, University of Sydney, Sydney 2006, Australia

**Keywords:** trampoline safety, child health, risk management, children’s play, injury prevention

## Abstract

Trampolining as an activity brings enjoyment and many health benefits, but at the same time it carries an injury risk. Most domestic trampoline users are children who are developing in skill, cognition, risk perception, physical strength and resilience to injury. Several common patterns of child trampoline injuries have been identified and countermeasures outlined in standards have been taken to reduce higher risk injury mechanisms, such as entrapment and falls from the trampoline through design, product and point of sale labelling. In Australia, the first national trampoline standard was published in 2003 which introduced improvements in trampoline design and requirements for labelling and padding. This work investigated the potential impact of these and subsequent changes based on almost two decades of emergency department trampoline injury data collected in Queensland, Australia. These data describe the changing representative proportion and pattern of trampoline injuries in Queensland over time by age, mechanism, gender, severity and nature of injury of injured persons up to the age of 14 years. The interrelationships between different injury characteristics were also analysed to propose the main factors influencing injury occurrence and severity. These findings seem to indicate that safety evolution in the form of enclosure nets, frame impact attenuation and entrapment protection have likely improved domestic trampoline safety. Other factors, such as adult supervision, minimum age and avoidance of multiple users, could further reduce injury but are harder to influence in the domestic setting.

## 1. Introduction

Trampolining is a popular sport that brings both fun and health benefits [1,2,3]. Trampolines can be designed in different shapes and sizes, and with various dynamic properties to suit different uses. Most domestic trampoline users are children, and this activity has been promoted to have numerous health benefits for them [4,5]. At the same time, trampolining is associated with injury risk to children [6,7,8] who may be less cognitively and physically adept and developed for the particular dynamics of this exercise. Injury risk may be heightened for children as they learn body coordination and synchronicity gradually throughout their life [9]. Injuries also arise through behavioural factors, such as collisions or ‘double-bouncing’ when multiple users are bouncing [10], and structural design issues that allow the user to fall from or become entrapped in the trampoline or to experience excessive trampoline forces [11,12].

Internationally each year, many children attend hospital emergency departments (EDs) as a result of injuries sustained while using trampolines [13,14,15]. While many of these injuries are self-limiting, such as sprains or strains that will generally recover with standard methods for relieving pain and swelling, head or spinal injuries that result in costly, debilitating and sometimes life-long damage to an individual’s neurological functioning are also seen [16,17].

In 1999, when domestic trampolines in Australia were becoming both popular and more affordable, an increase in serious injury cases was reported to Standards Australia, and a forum of leading experts convened in 2000 to discuss the development of a trampoline product standard. This forum consisted of a broad range of interest groups including the Australian Competition and Consumer Commission (ACCC), the Office of Fair Trading, Engineers Australia, the Australian Industry Group, the Department of Education and Training, the Department of Community Services, Gymnastics Australia, injury prevention experts and children’s play advocates, as well as all Australian trampoline manufacturers. Recommendation from the American Academy of Paediatrics to ‘ban the sale’ of domestic trampolines [18] was debated. It was ultimately agreed that a product safety standard would be written, specifying the minimum design requirements, product marking requirements and impact test methods for impact-attenuating padding for trampolines sold in Australia. As a result, Standards Australia established an expert technical committee to write Standard AS 4989:2003 Trampolines, which was published on 10 November 2003 [19].

In 2003 the only trampolines sold in Australia were rectangular, gymnastic grade products that provided the user with a ‘hard-feeling’ bounce experience, similar to that which they would experience at the local gymnasium. However, AS 4989:2003 was not written in a style that allowed technical product certification.

In or around 2006, there were major changes occurring in the Australian domestic trampoline industry, with rectangular trampolines being replaced by smaller round trampolines. Introducing the round trampoline onto the market allowed manufactures to install netted enclosures that, in principle, would prevent users from falling off the device [20,21]. Net enclosures aimed to reduce the main ‘fall off’ injury mechanism. The standard was therefore rewritten and on 3 October 2006 Standards Australia published AS 4989:2006 Trampolines—Safety Aspects [22].

The 2006 standard introduced an evidence-based impact-attenuation test method based on extensive research and testing by the University of Technology Sydney (UTS). In addition to netted enclosures, other design-mediated injury-prevention mechanisms, such as soft-edge protections, were being considered by the committee to reduce the ‘fall on’ trampoline injuries [23,24].

On 17 June 2015 AS 4989:2015 Trampolines for domestic use—Safety Aspects was published [25], which includes test methods for frame-edge impact attenuation, enclosure systems, containment and entrapment, structural integrity and protrusions. Extensive verification testing was undertaken by UTS before these test methods were adopted and published.

While standards are considered to be ‘living’ documents, able to dynamically reflect progress in science and technology as well as real-world systems, it is important to recognise that changes in standards and the industry in which they are contextualised, will have an impact on the user interaction and experience with the relevant product. It is therefore anticipated that the changes described in the AS 4989 standards for domestic trampoline safety, as well as the industry shifts over the past decades, will have resulted in changes to the epidemiology of trampoline injuries, such as their mechanism, nature, frequency, severity and location.

Injury prevention is best informed by an understanding of a whole system approach [26]. For domestic trampoline injury prevention, this includes consideration of the engineering and product standard aspects [27], contextualised by a temporal view of user developmental competency and behaviour and injury patterns. The objective of this study was to analyse almost two decades of injuries occurring from the use of domestic trampolines in the Australian state of Queensland, using population-level injury surveillance data [28]. Based on the analysis, this work also considered the potential impact on injury patterns from the introduction and usage of Australian standards that contributed to changes in the engineering design of these products throughout this study period.

## 2. Methods

In this population-level cohort study, we obtained emergency department (ED)-derived injury surveillance data from the Queensland Injury Surveillance Unit (QISU) for the 19-year study period—1 January 1999 to 31 December 2017. The state of Queensland, Australia is the nation’s second largest state, with a population of 5.3 million (2022), one-fifth of which is aged under 15 years. Around half of the population lives in the greater Brisbane metropolitan area, located in the south-east corner of the state. The remainder of the population is dispersed across an area of 1,727,000 square kilometres in several large regional metropolitan centres, numerous small towns and rural and farming communities [29]. QISU continuously collected injury surveillance data from participating EDs during the study period. Throughout the study period, participants attended 35 different hospitals for their treatment of trampoline-related injuries. Participating hospitals varied throughout the study period, though there were several long-term participating centres.

The secondary use of administrative data for injury surveillance has long been explored, despite the difficulty of obtaining accurate exposure information, a known limitation. Some attempts are more successful than others, where the narrative text provides additional data, for example concerning helmet wearing in a bicycle crash. Compared with admitted patient data, where injuries and external cause codes provide much more detail using the International Statistical Classification of Diseases codes [30], ED data do not use external cause codes, making accurate surveillance more challenging. The QISU data were utilised here by identifying cases from the narrative text data, whereby the text data indicated that the individual sustained an injury while on a trampoline and thereby defined a ’case’. What is unknown from these data, however, is the broader population denominator of ’all trampoline users’.

Data were collected by nurses at the point of patient triage via a system automated to open an ’injury module’ of data fields when the triage nurse ticked ’yes’ to an injury presentation on the electronic medical record. The injury module contained an injury narrative description and a minimum data set of coded fields, which were collated in accordance with the National Data Standards—Injury Surveillance [31]. A diagnosis code was added at the end of the ED stay by the treating medical practitioner. Data provided to QISU were then validated and stored within an SQL server database accessible by QISU staff only.

Data extraction and case validation for this study were completed by QISU staff using a combined free text and coded field search strategy to identify domestic trampoline-related injuries. The injury narrative descriptions of the extracted cases were then reviewed by two QISU coders to validate trampoline injuries and exclude non-related cases. The validated cases were then reviewed again by one of the authors to elicit additional non-coded information in relation to the injury event. The validated cases were classified into groups based on the injury narratives to reflect on the specific mechanisms, which included the following.

Fall mechanisms:
  - Fall from: a fall from the trampoline onto surrounding ground including child being propelled from the trampoline (from jump height) or from the trampoline height.
  - Fall through: a fall from the trampoline through the safety net, edge or the padding.
  - Fall on: a fall on trampoline (i.e., on the edge of the trampoline or on the padding) without being propelled off of the trampoline.

Non-fall mechanisms:
  - Collision: when an injured person has collided with part of the trampoline, a person or an object nearby while playing on the trampoline.
  - Entrapment/entanglement: when an injured person becomes entrapped in part of the trampoline (e.g., spring, net).
  - Over exertion: mechanical hyper extension causing strain or sprain, including a twisted ankle and hyper extension and hyper flexion of the neck.
  - Fall not elsewhere classified (NES): an unspecified falling mechanism.

When there are multiple mechanisms involved in an injury event, the mechanisms are categorised into primary and subsequent mechanisms.

Triage classifications were recorded by nurses at the point of presentation to the ED. These data comprised classifications in accordance with the Australasian Triage Scale (ATS), which is a clinical tool used to establish the maximum waiting time for medical assessment and treatment of a patient. The ATS is an ordinal scale from 1–5, where an ATS measurement of 1 demands that the patient must be immediately medically assessed and have treatment initiated, an ATS measurement of 2 demands that assessment takes place within 10 min, an ATS measurement of 3 demands that assessment takes place within 30 min, an ATS measurement of 4 demands that assessment takes place within 60 min and an ATS measurement of 5 demands that assessment takes place within 120 min.

Data were then exported from the QISU database into comma separated value files by secure server transfer, they were then imported into Matlab [32] for visualisation and analysis. Descriptive statistics were used to profile the injured cohort using a temporal view, to evaluate the injury patterns and frequency over time. Firstly, the injury data were presented demographically over the study period, to describe and explore the changing trends of the injury characteristics. The particulars of the injury epidemiology reported included number, age, gender, mechanism, nature, body region and severity. Interrelationships between different characteristics were analysed to evaluate the impact of associations.

## 3. Results

### 3.1. Demographic Distribution

During the almost 20-year study period, we identified 10,726 domestic trampoline-related injured individuals presented to hospital EDs in Queensland, Australia, with 10,353 of them were 14 years old or younger.

**Figure 1:**
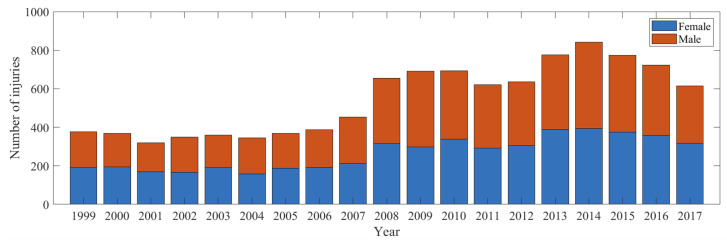
Number of injuries by sex between 1999 and 2017.

For these concerned 10,353 young injured individuals, the mean (standard deviation) age of them was 6.45 (3.34) years, with males representing 51.3% of all injuries. The number of domestic trampoline injuries by sex between 1999 and 2017 is shown in Figure 1. Injury numbers were highest in 2014, thereafter they decreased.

**Figure 2:**
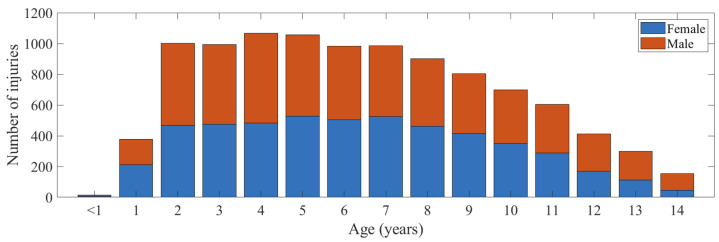
Number of injuries by age.

The number of injuries by age is shown Figure 2. Children aged between 2 and 7 years accounted for almost two-thirds (58.8%) of all cases.

### 3.2. Mechanism of Injury

**Figure 3:**
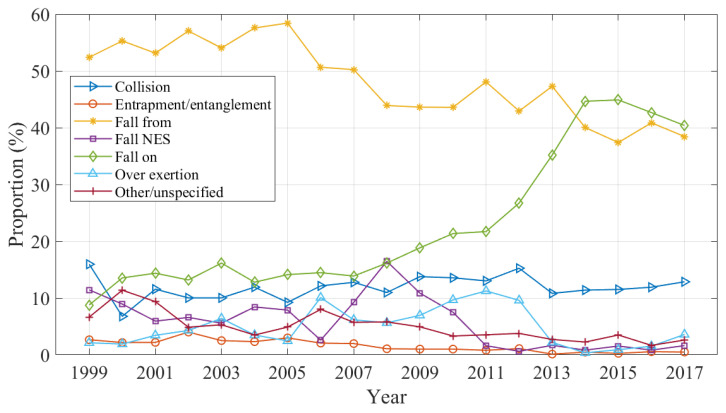
Proportion of injuries by mechanism.

Changes in the mechanism of injury are shown in Figure 3 as a proportion of all domestic trampoline-related injuries over the study period. Trampoline falls accounted for the largest proportion of cases over the study period (77.48%); however, there was a notable change in the mechanism of ’fall’. Falls from the trampoline decreased substantially after 2007, with higher proportions of ‘fall on’ injuries occurring simultaneously. There was also a small decrease in the proportion of entrapment injuries over the study period from the highest value of 4.01% in 2002 to the lowest value of 0.13% in 2013.

### 3.3. Nature of Injury

**Figure 4:**
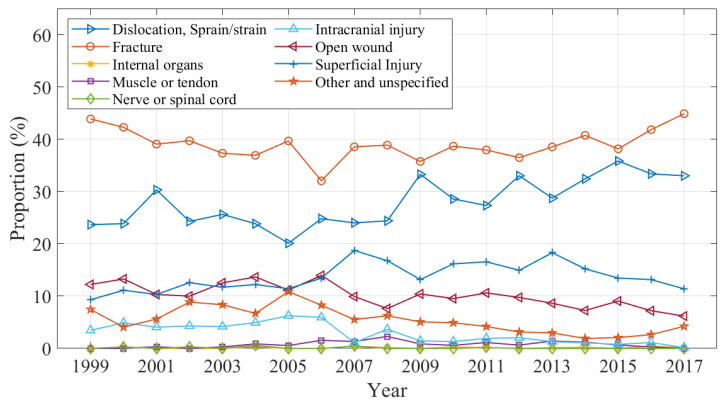
Proportion of injuries by nature.

Changes in the nature of injury over the study period is shown in Figure 4 as a proportion of all domestic trampoline-related injuries by year. The proportion of intracranial injuries declined from a peak of 6.25% in 2006 to 0.16% in 2017; notably, there was a steep decline in 2007 that remained consistent thereafter. Fractures and dislocations and sprain/strain injuries accounted for 39.06% and 28.95% of cases over the study period, respectively.

### 3.4. Body Region

**Figure 5:**
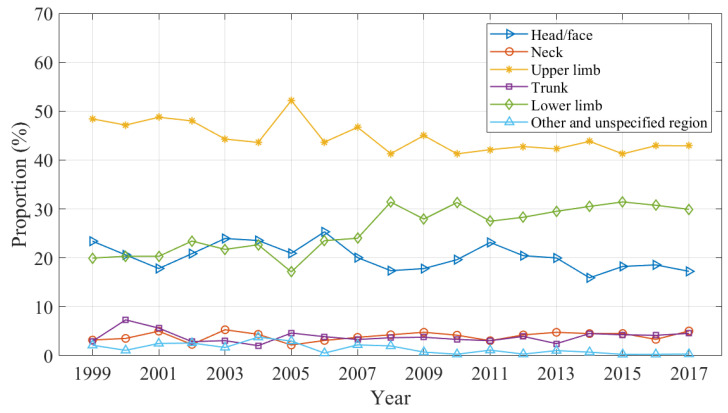
Proportion of injuries by body region.

Changes in the body region injured over the study period are shown in Figure 5 as a proportion of all domestic trampoline-related injuries by year. Upper limb, lower limb and head/face injures accounted for 43.99%, 28.18% and 19.74% of cases, respectively, over the study period. Figure 5 shows that head and face injury presentations were at a similar frequency to lower limb injury presentations up until around 2007; however the trajectory of head and face injuries declined after this time, which was simultaneous with the rise in lower limb injuries. The proportion of upper limb injuries showed a more gradual decline since around this same point.

### 3.5. Injury Severity

**Figure 6:**
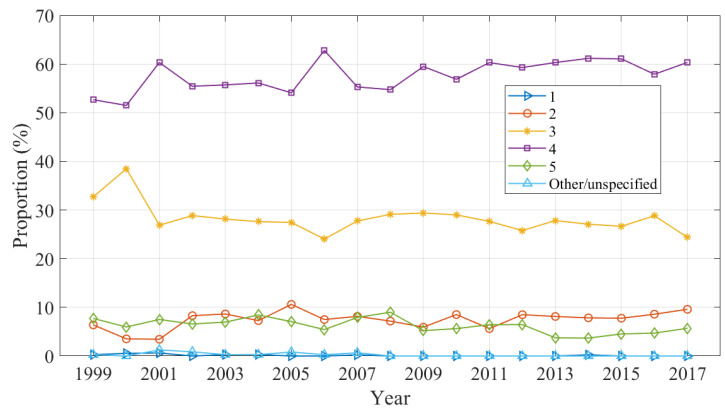
Proportion of injuries by severity.

Changes in the injury severity estimated by triage acuity is shown in Figure 6 as a proportion of all domestic trampoline-related injuries. Triage category 4 and 3 cases accounted for 58.16% and 28.08% of injuries across the study period, respectively. There was a small increase in category 2 cases early in the period.

### 3.6. Patterns of Injury Epidemiology and Mechanism

**Figure 7:**
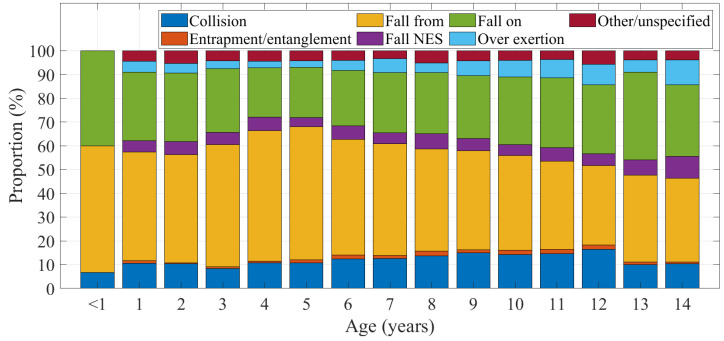
Injury mechanisms by age.

Injury mechanism varied somewhat by age and is shown in Figure 7 as a proportion of all domestic trampoline-related injuries. Children aged 4–5 years appeared more likely to experience ‘fall from’ injuries, whereas older aged children, around 10 years and over, appeared more likely to experience ‘fall on’ and over-exertion type injuries.

**Figure 8:**
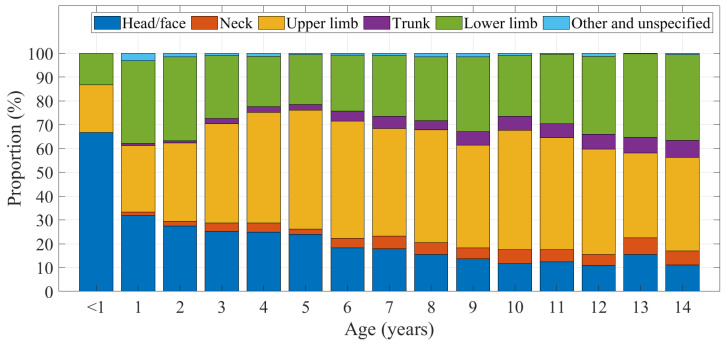
Injured body region by age.

In our study cohort, injury mechanisms varied by age, as did region of body injured while on a domestic trampoline. Toddler-aged children (around 2 years and under) experienced more head and face injuries than their older counterparts (Figure 8). Lower limb and trunk injuries appear with higher proportion among 12–14 year olds.

**Figure 9:**
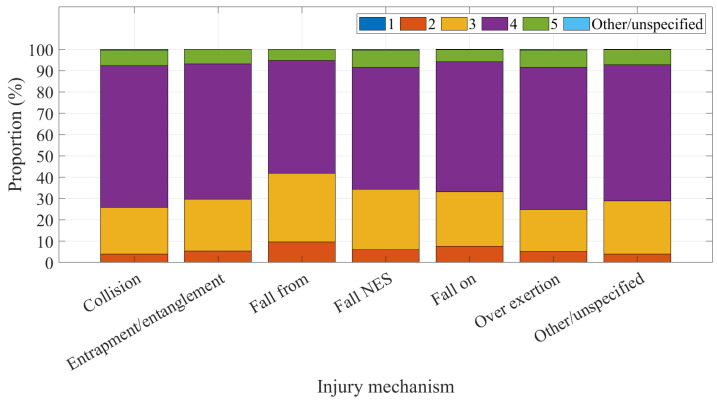
Injury severity by mechanism.

Changes in the injury severity, as estimated by triage acuity, is shown in Figure 9 as triage category proportion by injury mechanism. Falling from the trampoline was most likely to result in an assignment of triage category 2 or 3, compared with other mechanisms; proportions across the study period were 9.42% and 32.31%, respectively.

**Figure 10:**
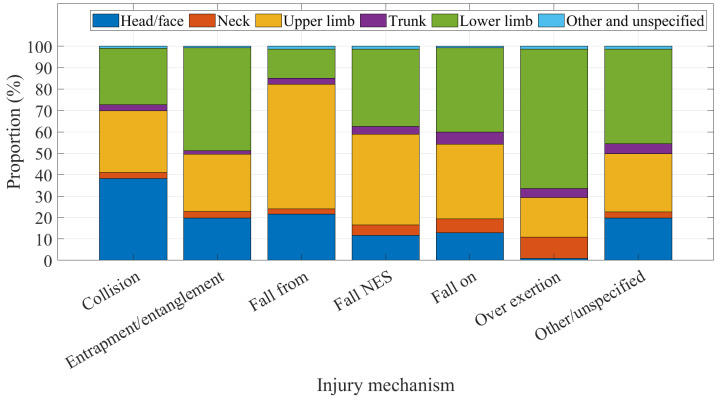
Injured body region by mechanism.

The body region that was injured varied by injury mechanism and is shown in Figure 10 as the proportion of injuries by mechanism. Collision had the highest proportion of head and face injuries (38.2%). Almost 60% (58.02%) of upper limb injuries resulted from falling from a trampoline, whereas the majority of lower limb injuries were ascribed to over exertion on a trampoline (65.05%).

## 4. Discussion

In this study of almost two decades of trampoline injuries that were recorded in a state-based injury surveillance system, we identified 10,726 trampoline-use-related injury episodes, and 10,353 of them were children and adolescents aged 14 years and younger.

The results presented demonstrate that falls from trampolines caused more severe injuries, supporting the necessity of trampoline enclosures. In these data from around 2006 onward, we saw the proportion of injuries due to falling off trampolines decline substantially. This coincided with the introduction of round trampolines into Australia—many with netting enclosures (a feature subsequently added to the published standard). However, this appeared to result in a concomitant rise in the proportion of ‘fall on’ trampoline-related injuries, implying that while the enclosures prevented players falling from trampolines, they rather fell onto the trampoline, or collided with simultaneous users.

The most common injuries experienced were dislocations, sprain/strains and fractures. To reduce dislocation and sprain/strain injuries, countermeasures could be considered that avoid over exertion, as around 40% of ‘fall on’ injuries and 70% of over exertion injuries were dislocation and sprain/strain injuries. The Australian Standard AS 4989 recognises, however, that a trampoline ideally provides an opportunity for gross motor skill development in users [33], which therefore demands a balance between risk and safety. The trampoline offers a unique opportunity to users, to develop coordination and balance as well as lower limb strength in articulating joints. AS 4989 does not therefore seek to remove all injury risk, ideally only serious injury risk should be removed. Around 45% of falls from trampolines and 35% of falls on trampolines resulted in fractures, which indicates that avoiding falls of both kinds could be an effective way to reduce fractures. For both fall-related and over exertion-related injuries, trampolines with lower stiffness could be expected to reduce the bouncing height, thus reducing the players’ reaction force when contacting with the trampoline [34].

The age distribution of injuries in the current study demonstrates that, proportionately, children from around 8 years old and over, are less represented in the cohort of injuries experienced (Figure 2). The potential reasons for this could be that older children have better skills and stronger bodies, which aids bouncing stability and, therefore, injury prevention. Children’s interest in trampolining could also decline with age when they have more choices for play activities [35,36]. Therefore, the lower proportions of older children among these data over time could represent lower participation of older children in trampolining activities.

Over this study we observed that among children around 1 to 4 years of age, the percentage of injuries caused by falls from trampolines increased somewhat, while injuries caused by falls on trampolines clearly decreased. Among children aged 5 to 12 years, the proportion of injuries caused by falls from trampolines decreased, while injuries caused by collisions and falls on trampolines increased. This could be explained by improved or matured management of coordination and stability on trampolines [37]. However, this age group may also attempt to perform more aggressive manoeuvres, such as jumping higher, somersaults etc, which increases the possibility of collision and ‘fall on’ injuries [38].

Our study was strengthened by the long time period and comprehensiveness of the data collection. This research also had some limitations. While the triage injury data fields have been in existence for many years now, the injury module fields are not mandatory, allowing triage nurses to skip some or all the injury fields, depending on clinical flow priorities. The completion rate of the injury module in QISU-participating hospitals varies monthly and averages at approximately 64%. The data collection methods and recording methodology have, however, been valid and consistent [39]. Injury information in the module, such as the mechanism of injury and location of injury, are also limited by the fact that the entry in this field can vary depending upon triaging style, clarity of information provided and clinical circumstances at the time when the nurse is attending to the injured person. When there is no documented information in the text field, injury cases will be assigned to ‘unspecified categories’. Furthermore, with the unavailable exposure data, i.e., the number of in-use trampolines and the population of trampoline users, it is not possible to calculate accurate rates that would more realistically reveal trends over time. This information is near impossible to collect, given the nature of backyard home use trampolines. We attempted to compensate for this by analysing proportions within the cohort collected over time and evaluating the various interrelationships within the epidemiology through a temporal lens that incorporated significant changes in product manufacturing, availability and standards.

## 5. Conclusions

This paper reviewed almost two decades of trampoline injury data collated by QISU from 1999 to 2017. During this period, numerous important changes occurred in Australia regarding domestic trampoline product safety, including the introduction of a national trampoline standard, modification of the trampoline shape and enclosure design, product labelling and geographic location of product manufacturing. The analysis of change in proportion (to manage the lack of accurate exposure data) to injury mechanism, nature, body region and severity over the years revealed how injury patterns changed during these years. Noting the enormity of such changes in a home-based activity, we propose that this indicates that the changes in trampoline manufacturing and standards influenced the injury conditions, which was reflected, therefore, in the injury data. The exploration of changes in interrelationships between different facets of trampoline injury epidemiology also seem to imply that the injury characteristics varied by participant age and activity mechanism. Our analyses infer that age restrictions and softer trampolines may further contribute to injury reduction. Additional insight into injury prevention efforts are recommended. The ideal study to evaluate these would be a whole-population cohort study, following all users of all trampolines over time, and reporting on exposure (trampoline use)-related injury incidents. This, however, would be an enormously challenging and costly study. Making injury data collection modules mandatory at the point of ED care, would assist in improving surveillance efforts. It is recommended that the current standard be revised and hazard mitigation interventions, such as self-closing and opening, be introduced. It is also recommended that compliance with the Trampoline Standard AS 4989 be mandated by the ACCC under Australian consumer law [40], and accompanying this, that the ACCC fund an Australia-wide education program on domestic trampoline safety.

## Data Availability

The data presented in this study are available on request from the corresponding author.

## Abbreviations

The following abbreviations are used in this manuscript:

ACCC	Australian Competition and Consumer Commission;
ATS	Australasian Triage Scale;
ED	emergency department;
NES	not elsewhere specified;
QISU	Queensland Injury Surveillance Unit;
UTS	University of Technology Sydney.
